# Student Satisfaction in Social Media–Based Learning Environments: Development, Validation, and Psychometric Evaluation of the CuSAERS (Questionnaire of Satisfaction With Educational Activities Performed on Social Media)

**DOI:** 10.2196/73805

**Published:** 2025-12-19

**Authors:** Roy La Touche, Álvaro Reina-Varona, Mónica Grande-Alonso, José Vicente León-Hernández, Joaquín Pardo-Montero, Néstor Requejo-Salinas, Raúl Ferrer-Peña, Alba Paris-Alemany

**Affiliations:** 1 Departamento de Fisioterapia, Centro Superior de Estudios Universitarios (CSEU) La Salle, Universidad Autónoma de Madrid Madrid Spain; 2 Motion in Brains Research Group, Centro Superior de Estudios Universitarios (CSEU) La Salle, Universidad Autónoma de Madrid Madrid Spain; 3 Instituto de Dolor Craneofacial y Neuromusculoesquelético (INDCRAN), Madrid, Spain. Madrid Spain; 4 Departamento de Cirugía, Ciencias Médicas y Sociales, Facultad de Medicina, Universidad de Alcalá, Alcalá de Henares, Spain. Alcalá de Henares Spain; 5 Clinical-Teaching Research Group on Rehabilitation Sciences (INDOCLIN), CSEU La Salle, UAM, Madrid, Spain Madrid Spain; 6 Hospital La Paz Institute for Health Research (IdiPAZ) Madrid Spain; 7 Cognitive Neuroscience, Pain and Rehabilitation Research Group (NECODOR), Faculty of Health Sciences, Rey Juan Carlos University, Alcorcón, Spain Alcorcón Spain; 8 Centro de Salud Entrevías, Gerencia Asistencial de Atención Primaria de la Comunidad de Madrid Madrid Spain; 9 Department of Basic Health Sciences, Universidad Rey Juan Carlos, Alcorcón, Spain. Alcorcón Spain

**Keywords:** academic engagement, collaborative learning, digital learning, educational technology, learning environments, psychometric validation, social media, student satisfaction

## Abstract

**Background:**

Social media platforms are increasingly integrated into higher education, enabling collaborative, student-centered learning. Yet, few instruments specifically measure students’ satisfaction with these activities across platforms. A brief, valid tool is needed to evaluate perceived quality and guide instructional design in social media–based learning environments.

**Objective:**

This study investigated the use of social media as educational tools in the university environment, with the aim of designing and validating the CuSAERS (Questionnaire of Satisfaction With Educational Activities Performed on Social Media).

**Methods:**

Using a mixed and sequential methodology, we explored the perceptions of bachelor’s and master’s degree students in physiotherapy who participated in teaching activities through X (formerly Twitter) and Instagram. The first phase of the project identified key dimensions of satisfaction from the literature, expert interviews, and cognitive interviews. The second phase assessed the psychometric properties of the CuSAERS in a sample of 150 students, addressing construct validity, internal reliability, concurrent validity, and discriminant validity.

**Results:**

Exploratory factor analysis supported a 3-factor structure—perception of learning, task satisfaction/environment, and self-realization—explaining 61.9% of the variance, with acceptable overall reliability. Concurrent validity was supported by moderate correlations with the Academic Satisfaction Scale. Master’s students reported higher scores than bachelor’s students.

**Conclusions:**

CuSAERS provides preliminary evidence as a promising measure of student satisfaction with social media–based learning activities; its use should remain formative and cautious until confirmatory and invariance analyses are completed.

**Trial Registration:**

No applicable.

## Introduction

Social media refers to online resources designed to facilitate interaction and engagement among individuals [1]. Over the last decade, these platforms have transformed the way people communicate, learn, and collaborate, extending far beyond personal and social contexts into education [2-4]. Tools such as X (formerly Twitter), Instagram, Facebook, and YouTube now support innovative pedagogical strategies aligned with digitalization and global collaboration, promoting learning experiences that transcend geographic and temporal boundaries [5-7].

In health professions education, social media can promote collaborative learning, reflective discussion, and peer interaction, enhancing students’ engagement and motivation [8-10]. Platforms like X facilitate professional dialogue and access to scientific information, while Instagram and YouTube enable visual learning and dissemination of clinical content [11,12]. However, drawbacks include information overload, variable content quality, and risks related to privacy or professionalism [13,14]. These limitations underscore the need for structured pedagogical design and critical evaluation of their educational use.

Despite the rapid adoption of social media in educational settings, systematic approaches to evaluate their educational impact remain limited. Existing measures often emphasize usability, frequency of use, or performance metrics while neglecting students’ affective responses and satisfaction with learning experiences [15-18]. Yet satisfaction is closely linked to engagement, retention, and perceived learning quality, making it a key outcome for educational quality assurance [19-21].

Assessing satisfaction requires specific, psychometrically sound instruments that capture the attitudinal evaluation of students toward learning activities conducted on social media, beyond usability or behavioral engagement [22,23]. Such measurement tools can inform educators and institutions about the perceived quality and effectiveness of teaching innovations in digital environments and guide continuous improvement in educational design [24].

This study aimed to develop and provide an initial psychometric evaluation of the CuSAERS (Questionnaire of Satisfaction With Educational Activities Performed on Social Media), a tool designed to assess students’ satisfaction with learning experiences delivered through social media. Specifically, we examined its internal consistency, factorial structure, and construct, concurrent, and discriminant validity across subgroups using different social media platforms.

## Methods

### Ethical Considerations

The university’s ethics committee of La Salle Higher Center for University Studies determined that, given the design of the study, formal ethics committee review and approval were not required and therefore granted an exemption, authorizing the study to be conducted without ethics committee approval. All students signed an informed consent document before participating, which explained the objectives of the study, the confidentiality of the data, and the voluntary nature of their participation. The database was encoded using an alphanumeric code, and no personal data was recorded. No compensation was provided to the participants.

### Study Design

This study used an exploratory sequential mixed design, combining qualitative and quantitative methods for the development, construction, and validation of the CuSAERS. The first qualitative phase, which included a literature review, semistructured interviews, expert content validation, cognitive interviews, and a pilot test, has been previously published [25]. This first phase had been aimed at identifying the dimensions of satisfaction perceived by students with regard to educational activities carried out on social media, such as interaction with the teacher, the quality of the content, and collaboration between colleagues.

The second phase focused on the psychometric evaluation of the CuSAERS. During this stage, the questionnaire was administered to a representative sample of bachelor’s and master’s students to assess its psychometric properties, including validity and reliability.

### Participants

The sample selected on a nonprobabilistic basis consisted of 160 bachelor’s and master’s degree students in physiotherapy from a Spanish university. Of these, 110 were bachelor’s students and 50 were master’s students. All participants were aged 18 years or older and enrolled in official academic programs. As an inclusion criterion, participants had to have previously participated in educational activities developed on social media, specifically on X or Instagram.

### Teaching Innovation Activities

The master’s students participated in an academic activity designed on Instagram. This activity consisted of the critical analysis of therapeutic exercises published in video format on this platform. Students watched the videos, identified technical errors, proposed modifications, and justified their proposals based on scientific evidence. This task not only fostered autonomous and critical learning but also promoted the use of social media as a professional learning tool. The students’ responses and observations were subsequently discussed in a digital forum supervised by the teacher, who acted as moderator and interlocutor, offering specific and targeted feedback.

The bachelor’s students participated in an activity developed on X. This activity included a virtual debate on aspects related to chronic pain. Each student had to perform prior research on the assigned topic and present their position in the form of X threads, using technical but accessible language. The debates were enriched with bibliographical references and the integration of specific hashtags to facilitate the monitoring of the discussions. The teacher’s involvement focused on moderating the debate, posing critical questions to deepen the arguments, and providing direct feedback on the content and quality of the interactions.

Both activities were designed to maximize students’ active participation, integrating the use of social media as an innovative pedagogical resource. The strategies used sought to foster critical, technical, and communicative skills in digital contexts, thus contributing to more dynamic and meaningful learning.

### Procedure

#### Phase 1: Development of CuSAERS

The first phase was described in a previous study and consisted of a literature review and interviews with experts in educational methodology, social media, and rehabilitation, as well as with physiotherapy students [25].

#### Phase 2: Psychometric Evaluation

In the second phase, the CuSAERS questionnaire was administered to a sample of 150 students, of whom 90 were bachelor’s and 60 were master’s students. The students completed the questionnaire online, and a cross-sectional design was used to assess the psychometric properties of the instrument.

### Data Analysis

#### Descriptive Statistics

The data analysis was performed exclusively using JAMOVI (version 2.6; The Jamovi Project) software, which allowed all statistical evaluations of the study to be performed. Descriptive statistics were used to summarize the categorical variables, expressed in absolute and relative frequencies, and the continuous variables, which were reported in terms of means, SDs, and 95% CIs.

#### Normality Assessment

The normality of the data was comprehensively assessed, using both statistical tests and graphical methods.

The Kolmogorov–Smirnov test was applied to determine whether the distributions of the variables differed significantly from a normal distribution. This test was complemented by a visual analysis using quantile–quantile (Q–Q) plots and histograms, which allowed the alignment of the data with the theoretical normal curve to be assessed.

In addition, skewness and kurtosis coefficients were calculated to assess the shape of the distributions.

Skewness values close to 0 indicate symmetric distributions, whereas positive or negative values indicate skewing to the right or left, respectively. As for kurtosis, values close to 3 indicate mesokurtic distributions, whereas higher or lower values suggest leptokurtic or platykurtic distributions, respectively. These metrics provided a quantitative framework for interpreting the normality of the data distributions.

Lastly, additional analyses were performed to assess the influence of outliers on the distributions. They were identified and visually examined using boxplots, which allowed us to determine whether these outliers had a significant impact on the structure of the data.

#### Construct Validity

We conducted an exploratory factor analysis (EFA) using the minimum residual extraction method with oblimin rotation. Sampling adequacy was evaluated with the Kaiser-Meyer-Olkin (KMO) index and Bartlett’s test of sphericity. The number of factors was determined using multiple criteria: Kaiser’s rule (eigenvalue ≥1), scree plot, parallel analysis, and exploratory graph analysis. Factors were retained when they had ≥2 items with minimal cross-loadings.

The root-mean-square error of approximation (RMSEA) was calculated with a 90% CI. RMSEA values up to 0.08 are considered indicative of a reasonable fit to the data, with values closer to 0.05 or lower suggesting a good fit. In addition, the Tucker-Lewis Index (TLI) was calculated, with values close to or above 0.95 indicating an excellent fit. The Bayesian information criterion (BIC) was also assessed, in which lower (more negative) values are preferred, suggesting a model that better explains the data with fewer parameters. Lastly, the model fit was confirmed using the chi-square test. A nonsignificant chi-square value indicates that the observed and expected covariances of the model do not differ significantly, supporting an adequate model fit.

Item retention followed a priori thresholds to preserve content validity and parsimony: only items with primary loadings ≥0.40 on a single factor were kept; items with cross-loadings within 0.20 of the primary loading or with communality/uniqueness values indicating poor common variance were considered for removal [26]. The final solution and retained items were based on these empirical criteria in conjunction with theoretical interpretability.

#### Internal Consistency (Reliability)

Internal consistency was assessed using Cronbach α coefficient, with values above 0.70 considered adequate, and McDonald omega (ω) coefficient, which provides a complementary estimate of internal consistency. This approach allowed a robust assessment of the homogeneity of the items that make up the dimensions of the CuSAERS.

#### Concurrent Validity

Concurrent validity was assessed by calculating Pearson correlations between CuSAERS scores and the Academic Satisfaction Scale (ASS), used as a reference instrument. The ASS defines academic satisfaction as “the well-being and enjoyment that students perceive in their experiences within the academic role” [27].

This instrument, composed of 7 items organized into a single factor, uses a 7-point Likert scale and has demonstrated high reliability (ordinal α=.92) and structural validity in Chilean university contexts [28].

In addition, previous experience in using social media, measured in months, was included as an additional variable to explore its relationship with CuSAERS scores. This information allowed us to assess whether the length of time spent using social media significantly influences the perception of academic satisfaction in activities performed in these environments.

The values of the correlations between the CuSAERS, ASS, and social media experience were interpreted according to the criteria of Schober et al [29]. The correlations were classified as follows: insignificant (0.00-0.10), weak (0.10-0.39), moderate (0.40-0.69), strong (0.70-0.89), and very strong (0.90-1.00).

#### Floor and Ceiling Effect

The presence of floor and ceiling effects was assessed by calculating the percentage of participants who obtained the lowest or highest possible scores on the questionnaire. An effect was considered significant if more than 15% of participants were at these extremes.

#### Discriminant Validity

The discriminant validity of the CuSAERS was explored by comparing groups of students with various levels of engagement in educational activities on social media platforms. Specifically, analyses were conducted to compare students who participated in activities on X versus those who used Instagram, examining differences in their satisfaction scores.

Additionally, students with prior experience using social media were compared with those without such experience to determine the impact of this variable on perceptions of educational satisfaction. A comparative analysis was also performed between physiotherapy master’s students and bachelor’s students to evaluate potential differences based on academic level.

The comparisons were conducted using the Mann–Whitney U test as a nonparametric method for independent samples, and effect sizes were calculated using rank-biserial correlation. Effect sizes were interpreted as small (*r*=0.10), moderate (*r*=0.30), and large (*r*=0.50). These analyses identified significant differences that support the ability of the CuSAERS to distinguish between groups with diverse educational characteristics and contexts.

Discriminant comparisons (eg, bachelor’s vs master’s degree; prior social media experience) are reported as exploratory contrasts with effect sizes and are not adjusted for covariates. In subsequent work we will conduct confirmatory factor analysis (CFA), multigroup measurement invariance (by degree level, gender, and age), cross-validation in an independent multisite sample, and covariate-adjusted models (eg, regression or standard error of means) to strengthen causal interpretability of group differences.

## Results

### Normality Analysis

The normality of the data was assessed through a comprehensive analysis that included both statistical tests and graphical methods, providing an in-depth understanding of the distribution of the items in the CuSAERS. The results of the Kolmogorov–Smirnov test indicated that the data significantly deviated from a normal distribution (*P*<.001), confirming that the variables did not meet the assumption of normality. This finding was consistent with skewness and kurtosis values, which suggested deviations from perfect symmetry and distributions far from the ideal mesokurtic shape. Most of the items showed negative skewness, indicating a tendency for participants to give higher scores on the satisfaction scale, while predominantly platykurtic kurtosis reflected lower concentration at the scale’s extremes.

The visual analysis complemented these statistical evaluations. Q–Q plots and histograms demonstrated that the data did not align with the theoretical normal curve, confirming the presence of biases and lighter tails in the distribution of scores. Additionally, the influence of outliers was explored using boxplots, which revealed greater dispersion in specific items, such as Item 8 and Item 12. These items, which had the highest SDs, reflected the heterogeneity in the participants’ perceptions regarding these specific aspects of the questionnaire.

### Descriptive Analysis

The descriptive analysis of the items showed means and medians centered around values close to 3, reflecting a tendency toward neutral or moderately positive responses. SDs ranged between 0.752 and 1.046, demonstrating differences in the dispersion of responses. Items with the highest dispersion were Item 8 and Item 12, whereas Item 16 presented the lowest variability.

Regarding distribution, most items exhibited negative skewness, suggesting a slight tendency toward higher scores, except for Item 8, which showed skewness close to 0. Platykurtic kurtosis predominated, indicating lower concentration of responses at the extremes, except for some items, such as Item 3 and Item 4, which displayed more leptokurtic distributions.

In terms of internal consistency, the item-total correlations were heterogeneous, ranging from −0.001 (Item 16) to 0.539 (Item 10). Items 11 and 16 stood out for having near-zero or negative item-total correlations, suggesting a lower contribution to the instrument’s consistency. Cronbach α and McDonald ω coefficients ranged between 0.645 and 0.716, and between 0.674 and 0.755, respectively, indicating moderate reliability overall. The highest reliability values were observed for Items 11 and 16, although these items had low item-total correlations, which might imply atypical behavior for these items (Table 1).

**Table 1:** Descriptive statistics and reliability coefficients upon item removals.

Item	Mean (SD)	Median (IQR)	Skewness	Kurtosis	Item 1: all correlation	Cronbach α	McDonald ω
Item 3	2.98 (0.797)	3.0 (3-3)	−0.948	1.01	0.481	0.653	0.683
Item 4	3.12 (0.764)	3.0 (3-4)	−0.977	1.31	0.455	0.657	0.685
Item 6	2.92 (0.821)	3.0 (3-3)	−0.897	0.7	0.296	0.679	0.729
Item 7	3.02 (0.846)	3.0 (3-4)	−0.552	−0.323	0.490	0.650	0.684
Item 8	2.49 (1.046)	3.0 (2-3)	−0.05	−1.18	0.287	0.683	0.732
Item 9	2.98 (0.831)	3.0 (2-4)	−0.352	−0.618	0.432	0.659	0.695
Item 10	3.19 (0.773)	3.0 (3-4)	−0.929	0.887	0.539	0.645	0.674
Item 11	3.18 (0.831)	3.0 (2-4)	−0.351	−1.47	0.029	0.715	0.752
Item 12	2.63 (1.05)	3.0 (2-3)	−0.204	−1.14	0.382	0.666	0.721
Item 13	3.04 (0.846)	3.0 (2-4)	−0.387	−0.78	0.274	0.682	0.731
Item 16	3.01 (0.752)	3.0 (2-4)	−0.021	−1.22	−0.001	0.716	0.755
Item 17	3.02 (0.935)	3.0 (3-4)	−0.786	−0.164	0.311	0.674	0.723

### Factor Analysis (Construct Validity)

The data were suitable for factor analysis (KMO=0.754; Bartlett’s *χ*²_45_=685; *P*<.001). An exploratory factor analysis with oblimin rotation supported a 3-factor solution explaining 61.9% of the variance (sum of squared loadings: 3.07, 1.56, 1.56; 30.7%, 15.6%, 15.6%). Interfactor correlations were small (*r*=0.06-0.20).

Factor 1 (perception of learning) showed high primary loadings for Item 10 (0.821), Item 4 (0.820), Item 3 (0.803), Item 7 (0.748), and Item 9 (0.695), with uniqueness ranging from 0.318 to 0.518 (Table 2).

**Table 2:** Factor loadings of the items in relation to the factor solution. Extraction was performed using the minimum residuals method with oblimin rotation. Only primary loadings ≥0.40 are displayed. Factor labels follow the retained 3-factor solution. Factor 3 should be interpreted with caution because of a boundary estimate for Item 8. Factor 2, representing task satisfaction/environment, was defined by Item 13 (0.763), Item 6 (0.745), and Item 17 (0.626), with higher uniqueness for Item 17 (0.593). Factor 3, representing self-realization, was defined by Item 8 (1.002; boundary estimate) and Item 12 (0.722).

Item	Statement	Factor 1 (perception of learning)	Factor 2 (task satisfaction)	Factor 3 (self-realization)	Uniqueness
Item 10	Training activities on social media foster my reflection, synthesis, and reasoning. [Las actividades formativas en redes sociales fomentaron mi reflexión, síntesis y razonamiento.]	0.821	—^a^	—	0.318
Item 4	Educational activities on social media motivate me to ask questions and participate in discussions. [Las actividades educativas en redes sociales me motivan a hacer preguntas y participar en discusiones.]	0.820	—	—	0.330
Item 3	Educational activities on social media promote my participation. [Las actividades educativas en redes sociales promueven mi participación.]	0.803	—	—	0.334
Item 7	The use of social media in education has increased my interest in the course content. [El uso de redes sociales en la educación aumentó mi interés en los contenidos de la asignatura.]	0.748	—	—	0.431
Item 9	Using social media as a learning tool is beneficial. [Es positivo usar redes sociales como herramienta de aprendizaje.]	0.695	—	—	0.518
Item 13	The time spent on training activities on social media is well utilized. [El tiempo dedicado a actividades formativas en redes sociales está bien aprovechado.]	—	0.763	—	0.419
Item 6	My educational experience on social media makes me feel that it is a suitable environment to express my ideas. [Mi experiencia educativa en redes sociales me hace sentir que es un entorno adecuado para expresar mis ideas.]	—	0.745	—	0.427
Item 17	My experience indicates that social media is suitable for acquiring knowledge related to my field of study. [Mi experiencia indica que las redes sociales son adecuadas para adquirir conocimientos relacionados con mi carrera.]	—	0.626	—	0.593
Item 8	I am satisfied with my participation in educational activities conducted through social media. [Estoy satisfecho con mi participación en las actividades educativas desarrolladas con redes sociales.]	—	—	1.002	0.001
Item 12	I am satisfied with what I have learned in educational activities on social media. [Estoy satisfecho con lo que he aprendido en las actividades educativas en redes sociales.]	—	—	0.722	0.441

^a^Empty cells indicate loadings <0.40 on that factor.

**Figure 1:**
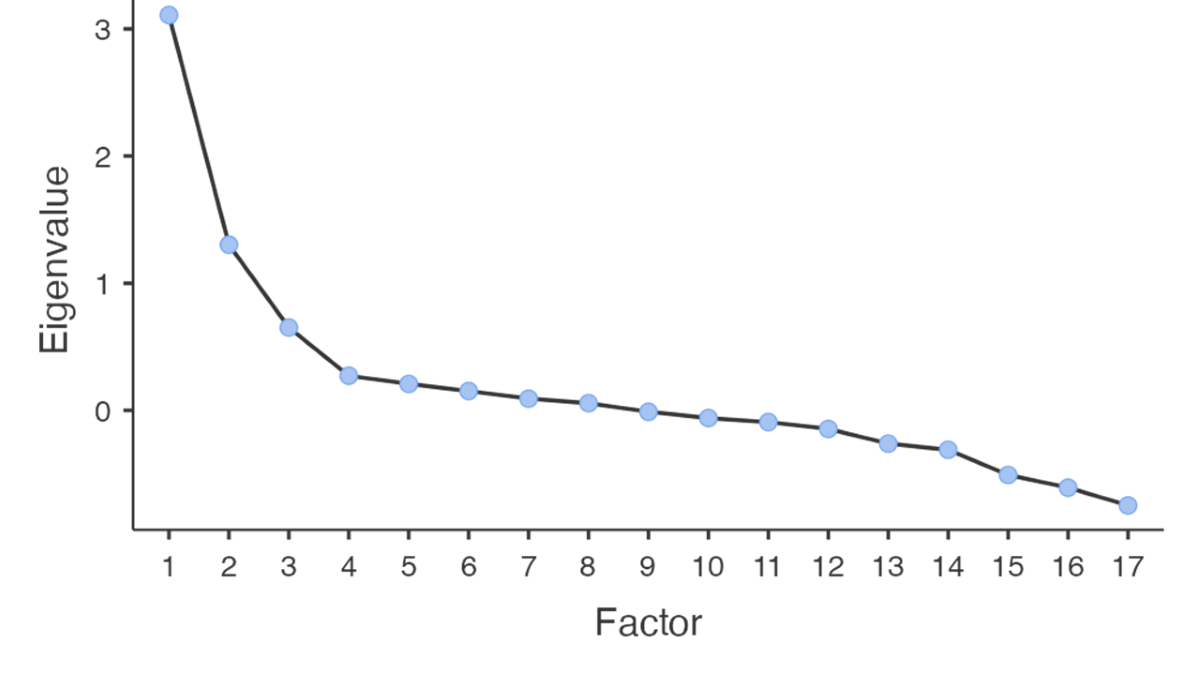
Scree plot of the exploratory factor analysis of the CuSAERS (Questionnaire of Satisfaction With Educational Activities Performed on Social Media), showing the eigenvalues of the extracted factors; the “elbow” in the curve suggests retaining 4 factors, which explain the majority of the total variance.

Overall model fit was acceptable (RMSEA=0.058; 90% CI 0.00-0.100; TLI=0.961; *χ*²_18_=27.9; BIC=–63.4; *P*=.06). All retained items met the a priori loading threshold (≥0.40), and cross-loadings were minimal (Table 2). The scree plot and parallel analysis supported the retention of three factors: the first 2 observed eigenvalues were clearly above the simulated distribution, the third was approximately at the simulated threshold, and from the fourth onward, the observed eigenvalues fell below the simulated ones (Figure 1). We note that Item 8 presented a boundary (Heywood) estimate—an occurrence that can arise in small samples and oblique solutions—so this third factor should be considered provisional pending confirmatory testing and item redevelopment in an independent, larger sample.

Consistent with the scree plot and parallel analysis (Figure 1), we retained the 3-factor solution as the primary model (Table 3). The 2-factor alternative, although parsimonious, collapses conceptually distinct domains and does not map cleanly onto the theorized constructs (Table S1 in Multimedia Appendix 1). The 4-factor alternative introduces a psychometrically weak structure: after applying the a priori retention rule (primary loading ≥0.40), one factor is left with a single indicator (Table S2 in Multimedia Appendix 1), which is not recommended for latent constructs. Taken together, the 3-factor model offers the best balance between empirical support and theoretical interpretability.

**Table 3:** Fit measures for the comparison between the 3 models. The 3-factor solution is the primary model; the 2- and 4-factor solutions are provided for comparison.

Model factors	Variance (%)	RMSEA^a^ (90% CI)	TLI^b^	BIC^c^	Chi-square (*df*)	*P* value
2	57.5	0.064 (<0.001 to 0.111)	0.963	−44.4	21.6 (13)	.06
3	61.9	0.058 (<0.001 to 0.1)	0.961	−63.4	27.9 (18)	.06
4	58.4	0.055 (<0.001 to 0.092)	0.948	−86	35.8 (24)	.06

^a^RMSEA: root-mean-square error of approximation.

^b^TLI: Tucker-Lewis Index.

^c^BIC: Bayesian information criterion.

### Internal Consistency

Table 4 presents the descriptive statistics and reliability coefficients for the evaluated scales. The overall mean for the CuSAERS was 2.93 (SD 0.503), with moderate internal consistency coefficients for both Cronbach α=0.743 and McDonald ω=0.787. Among the subscales, perception of learning showed the highest internal consistency (α=0.882; ω=0.884) and a mean of 3.06 (SD 0.662), indicating a positive perception of learning.

**Table 4:** Descriptive statistics and reliability of the CuSAERS (Questionnaire of Satisfaction With Educational Activities Performed on Social Media) questionnaire and its subscales.

Scale	Mean (SD)	Cronbach α	McDonald ω
CuSAERS total	2.93 (0.503)	0.743	0.787
Perception of learning	3.06 (0.662)	0.882	0.884
Task satisfaction	2.99 (0.709)	0.749	0.756
Self-realization	2.56 (0.977)	0.849	0.849

### Concurrent Validity

Concurrent validity was assessed using Spearman correlations between the CuSAERS scores and the ASS, as well as prior experience with social media (Table 5). The results showed that the total CuSAERS score had a moderate positive correlation with the ASS (ρ=0.59; *P*<.001), supporting its validity as an instrument to evaluate the perception of educational activities on social media. Additionally, significant positive correlations were observed between the ASS and the subscales perception of learning (ρ=0.44; *P*<.001), task satisfaction (ρ=0.31; *P*<.001), and self-realization (ρ=0.29; *P*<.01), indicating a consistent relationship between academic satisfaction and the dimensions evaluated by the CuSAERS.

**Table 5:** Correlation matrix.

Variable	ASS^a^	Social media experience	CuSAERS^b^ total	Perception of learning	Task satisfaction	Self-realization
ASS	—^c^					
Social media experience	0.17^d^	—				
CuSAERS total	0.59^e^	0.27^e^	—			
Perception of learning	0.44^e^	0.20^f^	0.72^e^	—		
Task satisfaction	0.31^e^	0.14	0.54^e^	0.09	—	
Self-realization	0.29^f^	0.23^f^	0.56^f^	0.10	0.19^d^	—

^a^ASS: Academic Satisfaction Scale.

^b^CuSAERS: Questionnaire of Satisfaction With Educational Activities Performed on Social Media.

^c^Not applicable.

^d^*P*<.05.

^e^*P*<.001.

^f^*P*<.01.

**Figure 2:**
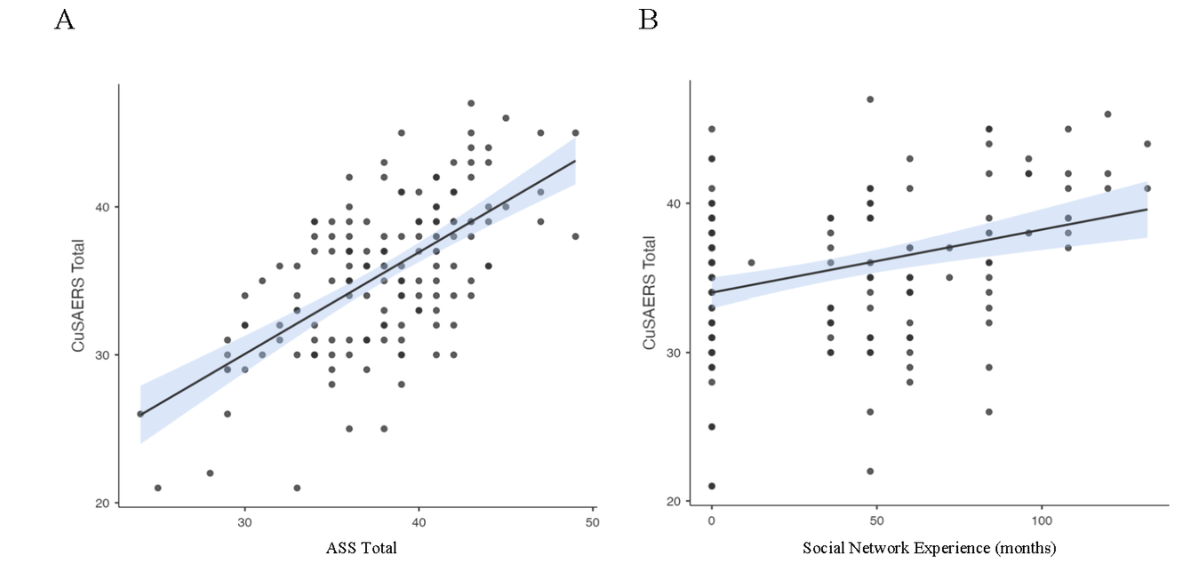
Correlations between the total CuSAERS (Questionnaire of Satisfaction With Educational Activities Performed on Social Media) score and external variables, showing (A) the relationship with Academic Satisfaction Scale (ASS) and (B) the relationship with experience with social media (measured in months).

On the other hand, prior experience with social media showed a weak positive correlation with the CuSAERS total score (ρ=0.27, *P*<.001), as well as with perception of learning (ρ=0.20; *P*<.01) and self-realization (ρ=0.23; *P*<.01). However, no significant correlations were observed with the task satisfaction subscale (ρ=0.14; *P*>.05). These results suggest that, although prior experience with social media has a positive relationship with overall perception and some specific aspects evaluated by the CuSAERS, its influence on dimensions such as motivation is limited (Figure 2).

### Discriminant Validity

**Figure 3:**
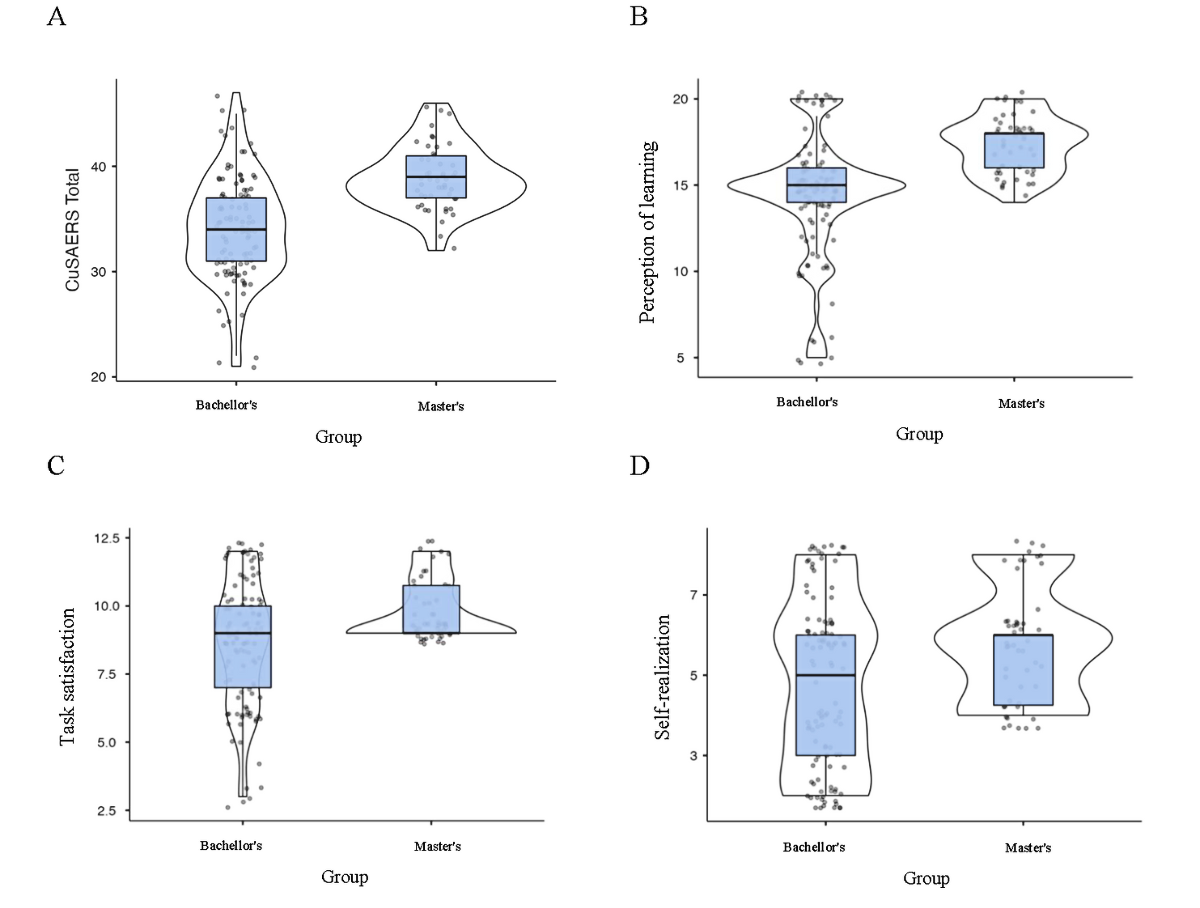
Comparative box and violin plots between bachelor’s and master’s groups in the CuSAERS (Questionnaire of Satisfaction With Educational Activities Performed on Social Media) subscales and total score, showing (A) total CuSAERS scores, (B) the perception of learning subscale, (C) the task satisfaction subscale, and (D) comparison of the self-realization subscale.

The discriminant validity of the CuSAERS was evaluated by comparing groups of students based on their academic level (bachelor’s or master’s). These comparisons were conducted using the nonparametric Mann-Whitney *U* test, and the effect size was calculated using rank-biserial correlation. The statistical results and graphical inspection through box and violin plots reveal clear differences between the 2 groups, supporting the CuSAERS’s ability to discriminate between students with different educational levels (Figure 3).

For the total CuSAERS score (Figure 3A), master’s students had significantly higher scores (*U*=1113; *P*<.001), with a median of 39 (IQR 37-41), compared with the bachelor’s group, whose median was 34 (IQR 31-37). Box and violin plots showed greater dispersion in the bachelor’s group, whereas scores for the master’s group were more compact and centered on higher values.

The perception of learning subscale (Figure 3B) also showed significant differences (*U*=1064; *r*=0.613; *P*<.001), with higher scores and less dispersed distributions in the master’s group (median 18, IQR 16-18). The plots reveal greater variability in the bachelor’s group, with individual cases at considerably low levels.

For the task satisfaction (Figure 3C) and self-realization (Figure 3D) subscales, significant differences were found (*U*=2006; *r*=0.271; *P*=.005). For task satisfaction, the master’s group showed higher scores with less dispersion (median 9, IQR 9-10.8), whereas for self-realization, the master’s group’s scores were also higher (median 6, IQR 4.25-6), in contrast to the greater dispersion and lower values observed in the bachelor’s group (Table 6).

**Table 6:** Comparison of CuSAERS scores by academic level.

Variable	Bachelor’s degree (physiotherapy; n=110)				Master’s degree (physiotherapy; n=50)				Mann-Whitney *U* test		*P* value		Effect size
	Mean (SD)	Median (IQR)	SEM^a^	Mean (SD)		Median (IQR)	SEM						
CuSAERS Total	34.10 (4.96)	34 (31-37)	0.47	38.96 (3)		39 (37-41)	0.42	1113		<.001		0.595	
Perception of learning	14.86 (3.46)	15 (14-16)	0.33	17.34 (1.62)		18 (16-18)	0.23	1064		<.001		0.613	
Task satisfaction	8.62 (2.37)	9 (7-10)	0.22	9.78 (0.15)		9 (9-10.8)	0.15	2006		.005		0.271	
Self-realization	4.81 (2.08)	5 (3-6)	0.19	5.80 (1.44)		6 (4.25-6)	0.20	2006		.005		0.271	

^a^SEM: standard error of means.

The results also indicated differences between students with and without prior experience in social media. The group with experience obtained higher scores across all subscales and in the total CuSAERS score, although the differences were only significant for the total score (*U*=2514; *r*=0.2045; *P*=.03). For the perception of learning and self-realization, although statistical significance was not reached (*P*>.05), trends toward higher scores were observed in the group with experience (Table 7).

**Table 7:** Comparison of CuSAERS (Questionnaire of Satisfaction With Educational Activities Performed on Social Media) scores by participants’ experience with social media.

Variable	Experience with social media (n=89)				No experience with social media (n=71)				Mann-Whitney *U* test		*P* value		Effect size
	Mean (SD)	Median (IQR)	SEM^a^	Mean (SD)		Median (IQR)	SEM						
CuSAERS Total	36.42 (4.96)	37 (33-39)	0.53	34.55 (4.77)		35 (31-38)	0.57	2514		.03		0.205	
Perception of learning	15.65 (2.97)	15 (15-17)	0.31	14.85 (3.67)		15 (14-17.5)	0.44	2794		.20		0.116	
Task satisfaction	9.06 (2.31)	9 (8-11)	0.25	8.89 (1.89)		9 (8-10)	0.22	2920		.40		0.076	
Self-realization	5.35 (2.09)	6 (4-7)	0.22	4.83 (1.74)		4 (4-6)	0.21	2639		.07		0.165	

^a^SEM: standard error of means.

Results for the motivation facet—considered at the content-validation stage but not retained by the final EFA—are provided in Table S3 in Multimedia Appendix 1 for completeness. These results suggest that familiarity with the use of social media might positively influence overall perceptions of educational activities, although its impact on specific subdimensions is more limited.

## Discussion

### Overview

The present findings support CuSAERS as a preliminary instrument for assessing student satisfaction in social media–mediated learning environments. In its 3-factor configuration—perception of learning, task satisfaction/environment, and self-realization—the scale shows acceptable reliability at the total-score level and coherent relations with external criteria, while some facets remain candidates for refinement. Construct validity, analyzed through EFA, supported a 3-factor solution that aligns with prior conceptualizations of satisfaction in digital educational settings [18,19] and with the multidimensional notion of academic satisfaction [30-32].

Among the 3 identified factors, perception of learning and self-realization showed the strongest relations with external criteria, consistent with previous findings highlighting the importance of acquiring new skills and the sense of achievement in digital learning experiences [5,7]. These components moderately correlated with the ASS, supporting the concurrent validity of the CuSAERS. In line with research linking satisfaction to performance and retention, students who perceived greater learning and personal growth also tended to report higher overall academic satisfaction [33,34]. Socioemotional elements and social support are key in online contexts; recent work shows that emotional support and effective communication through social media increase student satisfaction and motivation [35].

Psychometrically, internal consistency was acceptable overall. The perception of learning dimension exhibited the highest internal consistency, reflecting its stability. Although motivation emerged at the content-validation stage as a relevant facet, the present exploratory analyses did not retain a stable motivation factor. This facet remains a target for item redevelopment and confirmatory evaluation in future studies. Notably, recent studies suggest that visually rich platforms (eg, Instagram) can enhance motivation, whereas text-centric platforms (eg, X) may require higher digital literacy for comparable satisfaction [36,37]. These considerations are congruent with standard scale-development practice, where less stable content is refined after initial validation [26].

Discriminant analyses revealed significant differences between bachelor’s and master’s students, with higher scores among the latter on most subscales. Explanations may include greater academic and professional experience and stronger self-management skills among master’s students [3,23].

By contrast, bachelor’s students engaged in an X-based debate on chronic pain, a context in which lower academic maturity and less experience with educational uses of social media may temper satisfaction [2,9,15,38-41]. Importantly, the bachelor group’s scores were moderately positive, indicating overall acceptance of social media–based activities, while calling for replication in other health disciplines.

Regarding platform differences (X vs Instagram), platform use was not randomized and tasks differed in nature; therefore, any platform contrasts are descriptive and noncausal. Prior literature suggests that Instagram’s audiovisual format may favor motivation and participation [8,42,43], whereas X is powerful for debate but may demand greater digital literacy [11,16].

The moderate correlation between CuSAERS scores and ASS scores further supports the instrument’s concurrent validity, indicating that satisfaction with social media activities is related to overall academic satisfaction [27,28]. Prior experience with social media showed weak-to-moderate associations with satisfaction, suggesting that technological familiarity helps but does not solely determine satisfaction; interaction quality, content relevance, social presence, and the teacher’s facilitating role also matter [13,14,19,21,36,44].

Taken together, the findings support CuSAERS as a preliminary instrument with acceptable reliability at the total-scale level and a provisional self-realization subscale. The scale can inform formative pedagogical reflections, whereas policy or high-stakes decisions should await confirmatory replication and measurement-invariance testing.

### Limitations and Future Perspectives

This study used a nonprobabilistic, single-institution sample, which limits generalizability [45]. Future work should include larger, multisite samples spanning diverse programs and contexts [46,47]. Platform use was not randomized: students in the master’s degree used Instagram and students in the bachelor’s degree used X, and activities differed, so platform effects are confounded by academic level and task; future studies will randomize platform assignment or use within-subject counterbalanced designs with covariate-adjusted models.

Next steps will subject the 3-factor structure to CFA, test configural, metric, and scalar measurement invariance (degree level, gender, age), and conduct cross-validation in an independent, multi-institutional sample [48]. The motivation facet will undergo item redevelopment prior to confirmatory testing. Beyond psychometrics, it will be useful to relate CuSAERS to outcomes such as academic performance, motivation types, peer and teacher interaction, and participation in learning communities [49].

### Conclusions

This study provides initial evidence that the CuSAERS is a promising, early-stage instrument for assessing student satisfaction with learning activities delivered over social media. In its 3-factor configuration—perception of learning, task satisfaction/environment, and self-realization—the scale showed acceptable reliability at the total-score level and coherent associations with an established academic satisfaction measure. Group contrasts by academic level were observed, but platform-related differences should be interpreted cautiously given the nonrandomized design.

CuSAERS should be regarded as an early-stage measure with promising properties. Its routine use will benefit from CFA, measurement-invariance testing, and multisite replication, alongside ongoing item refinement. Within the broader trend toward digitization in higher education, tools such as CuSAERS can inform formative pedagogical decisions and help tailor social media–based activities to student needs, while definitive applications should await confirmatory evidence.

## Appendix

Multimedia Appendix 1 Suuplementary tables containing descriptive statistics and reliability coefficients upon item removal, two-factor EFA solution, and four-factor EFA solution.

## Abbreviations

ASS: Academic Satisfaction Scale
BIC: Bayesian information criterion
CFA: confirmatory factor analysis
CuSAERS: Questionnaire of Satisfaction With Educational Activities Performed on Social Media
EFA: exploratory factor analysis
KMO: Kaiser-Meyer-Olkin
Q–Q: quantile–quantile
RMSEA: root-mean-square error of approximation
TLI: Tucker-Lewis Index
