# Supplementary material for: Student Satisfaction in Social Media–Based Learning Environments: Development, Validation, and Psychometric Evaluation of the CuSAERS (Questionnaire of Satisfaction With Educational Activities Performed on Social Media)
Source: JMIR Med Educ. 2025 Dec 19;11:e73805. doi: 10.2196/73805 (PMC12759299; doi:10.2196/73805)
Supplement: Multimedia Appendix 1 [file mededu_v11i1e73805_app1.docx]

**Supplementary Material.**

**Table S1.** Descriptive statistics and reliability coefficients upon item removal. This table shows the descriptive statistics of all the analyzed items before the factorial analysis.

| **Item** | **Mean** | **Standard Deviation** | **Median** | **Assymetry** | **Kurtosis** | **Item 1-all correlation** | **Cronbach’s** **alfa (α)** | **McDonald’s omega (ω)** |
| --- | --- | --- | --- | --- | --- | --- | --- | --- |
| **Item 1** | 2,82 | 0,951 | 3,0 | -0,34 | -0,826 | 0,056 | 0,642 | 0,666 |
| **Item 2** | 3,14 | 0,487 | 3,0 | 0,346 | 0,737 | -0,004 | 0,635 | 0,673 |
| **Item 3** | 2,98 | 0,797 | 3,0 | -0,948 | 1,01 | 0,417 | 0,588 | 0,607 |
| **Item 4** | 3,12 | 0,764 | 3,0 | -0,977 | 1,31 | 0,408 | 0,59 | 0,606 |
| **Item 5** | 3,26 | 0,74 | 3,0 | -0,463 | -1,05 | 0,004 | 0,641 | 0,673 |
| **Item 6** | 2,92 | 0,821 | 3,0 | -0,897 | 0,7 | 0,316 | 0,602 | 0,64 |
| **Item 7** | 3,02 | 0,846 | 3,0 | -0,552 | -0,323 | 0,47 | 0,578 | 0,599 |
| **Item 8** | 2,49 | 1,046 | 3,0 | -0,05 | -1,18 | 0,303 | 0,602 | 0,642 |
| **Item 9** | 2,98 | 0,831 | 3,0 | -0,352 | -0,618 | 0,408 | 0,588 | 0,61 |
| **Item 10** | 3,19 | 0,773 | 3,0 | -0,929 | 0,887 | 0,521 | 0,574 | 0,59 |
| **Item 11** | 3,18 | 0,831 | 3,0 | -0,351 | -1,47 | -0,005 | 0,646 | 0,673 |
| **Item 12** | 2,63 | 1,05 | 3,0 | -0,204 | -1,14 | 0,409 | 0,582 | 0,628 |
| **Item 13** | 3,04 | 0,846 | 3,0 | -0,387 | -0,78 | 0,266 | 0,609 | 0,646 |
| **Item 14** | 3,25 | 0,604 | 3,0 | -0,173 | -0,524 | 0,109 | 0,627 | 0,66 |
| **Item 15** | 3,08 | 0,955 | 3,0 | -0,371 | -1,36 | 0,025 | 0,646 | 0,669 |
| **Item 16** | 3,01 | 0,752 | 3,0 | -0,021 | -1,22 | -0,016 | 0,644 | 0,675 |
| **Item 17** | 3,02 | 0,935 | 3,0 | -0,786 | -0,164 | 0,311 | 0,601 | 0,637 |

|  |
| --- |

**Table S2.** Two-factor EFA solution (primary loadings ≥ 0.40).

| Item | Statement | Factor 1 Perception of learning | Factor 2 Task satisfaction | Uniqueness |
| --- | --- | --- | --- | --- |
| Item 10 | Training activities on social media foster my reflection, synthesis, and reasoning.  Las actividades formativas en redes sociales fomentaron mi reflexión, síntesis y razonamiento. | 0.824 |  | 0.316 |
| Item 4 | Educational activities on social media motivate me to ask questions and participate in discussions.  Las actividades educativas en redes sociales me motivan a hacer preguntas y participar en discusiones. | 0.811 |  | 0.342 |
| Item 3 | Educational activities on social media promote my participation.  Las actividades educativas en redes sociales promueven mi participación. | 0.806 |  | 0.350 |
| Item 7 | The use of social media in education has increased my interest in the course content.  El uso de redes sociales en la educación aumentó mi interés en los contenidos de la asignatura. | 0.748 |  | 0.429 |
| Item 9 | Using social media as a learning tool is beneficial.  Es positivo usar redes sociales como herramienta de aprendizaje. | 0.695 |  | 0.518 |
| Item 13 | The time spent on training activities on social media is well utilized.  El tiempo dedicado a actividades formativas en redes sociales está bien aprovechado. |  | 0.774 | 0.402 |
| Item 6 | My educational experience on social media makes me feel that it is a suitable environment to express my ideas.  Mi experiencia educativa en redes sociales me hace sentir que es un entorno adecuado para expresar mis ideas. |  | 0.747 | 0.443 |
| Item 17 | My experience indicates that social media is suitable for acquiring knowledge related to my field of study.  Mi experiencia indica que las redes sociales son adecuadas para adquirir conocimientos relacionados con mi carrera. |  | 0.615 | 0.599 |

Model fit (two-factor): RMSEA = 0.064 (90% CI 0.00–0.111), TLI = 0.963, χ²(13) = 21.6, p = .062; KMO = 0.811; Bartlett’s χ²(28) = 537, p < .001.

**Table S3.** Four-factor EFA solution (primary loadings ≥ 0.40).

| Item | Statement | Factor 1 Perception of learning | Factor 2 Task satisfaction | Factor 3 Self-realization | Factor 4 Motivation | Uniqueness |
| --- | --- | --- | --- | --- | --- | --- |
| Item 10 | Training activities on social media foster my reflection, synthesis, and reasoning.  Las actividades formativas en redes sociales fomentaron mi reflexión, síntesis y razonamiento. | 0.822 |  |  |  | 0.318 |
| Item 4 | Educational activities on social media motivate me to ask questions and participate in discussions.  Las actividades educativas en redes sociales me motivan a hacer preguntas y participar en discusiones. | 0.816 |  |  |  | 0.313 |
| Item 3 | Educational activities on social media promote my participation.  Las actividades educativas en redes sociales promueven mi participación. | 0.805 |  |  |  | 0.334 |
| Item 7 | The use of social media in education has increased my interest in the course content.  El uso de redes sociales en la educación aumentó mi interés en los contenidos de la asignatura. | 0.750 |  |  |  | 0.431 |
| Item 9 | Using social media as a learning tool is beneficial. Es positivo usar redes sociales como herramienta de aprendizaje. | 0.699 |  |  |  | 0.516 |
| Item 13 | The time spent on training activities on social media is well utilized. El tiempo dedicado a actividades formativas en redes sociales está bien aprovechado. |  | 0.779 |  |  | 0.393 |
| Item 6 | My educational experience on social media makes me feel that it is a suitable environment to express my ideas.  Mi experiencia educativa en redes sociales me hace sentir que es un entorno adecuado para expresar mis ideas. |  | 0.733 |  |  | 0.426 |
| Item 17 | My experience indicates that social media is suitable for acquiring knowledge related to my field of study.  Mi experiencia indica que las redes sociales son adecuadas para adquirir conocimientos relacionados con mi carrera. |  | 0.625 |  |  | 0.592 |
| Item 8 | I am satisfied with my participation in educational activities conducted through social media.  Estoy satisfecho con mi participación en las actividades educativas desarrolladas con redes sociales. |  |  | 1.003 |  | 0.002 |
| Item 12 | I am satisfied with what I have learned in educational activities on social media.  Estoy satisfecho con lo que he aprendido en las actividades educativas en redes sociales. |  |  | 0.721 |  | 0.436 |
| Item 16 | Educational activities on social media increase my motivation to learn more than traditional methods.  Las actividades educativas en redes sociales aumentan mi motivación para aprender más que con los métodos tradicionales. |  |  |  | 0.850 | 0.277 |

Model fit (four-factor): RMSEA = 0.055 (90% CI 0.00–0.092), TLI = 0.948, χ²(24) = 35.8, p = .057; KMO = 0.730; Bartlett’s χ²(66) = 707, p < .001. Note: Factor 3 is labeled ‘Self-realization’; Factor 4 as ‘Motivation’.
